# Lung Infection by Human Bocavirus Induces the Release of Profibrotic Mediator Cytokines *In Vivo* and *In Vitro*

**DOI:** 10.1371/journal.pone.0147010

**Published:** 2016-01-25

**Authors:** Soumaya Khalfaoui, Vivien Eichhorn, Christian Karagiannidis, Inga Bayh, Michael Brockmann, Monika Pieper, Wolfram Windisch, Oliver Schildgen, Verena Schildgen

**Affiliations:** 1 Kliniken der Stadt Köln gGmbH, University Hospital Witten/Herdecke, Cologne-Merheim, Ostmerheimer Strasse 200, Institute for Pathology, D-51109 Cologne, Germany; 2 Kliniken der Stadt Köln gGmbH, University Hospital Witten/Herdecke, Cologne-Merheim, Ostmerheimer Strasse 200, Department of Pneumology and Critical Care Medicine, D-51109 Cologne, Germany; 3 Institute for Medical Biometry and Epidemiology, Faculty of Health, Alfred-Herrhausen-Str. 50, Witten/Herdecke University, Witten, Germany; Faculty of Biochemistry Biophysics and Biotechnology, Jagiellonian University, POLAND

## Abstract

Human Bocavirus subtype 1 (HBoV1) is associated with respiratory diseases and may contribute to chronic lung diseases by persisting in the infected host. Here the question was addressed if HBoV infections could contribute to fibrogenesis processes as suggested by previously published clinical observations. Cytokine profiles induced by HBoV infection in CuFi-8 air-liquid interphase cell cultures and in bronchoalveolar lavage fluid (BALF) of 20 HBoV-positive and 12 HBoV-negative patients were analysed by semi-quantitative Western spot blot analyses. Although lots of cytokines were regulated independently of HBoV status, several cytokines associated with lung fibrosis and tumour development, e.g., EGF, VEGF, TARC (CCL17), TNF-α, TNF-β, TIMP-1, were clearly upregulated in the HBoV-positive cohort. These findings suggest that the development of lung fibrosis might be triggered by HBoV induced cytokine expression.

## Introduction

The human bocavirus (HBoV) is the fourth most common virus detected in respiratory infections [1] and the second autonomous human parvovirus causing respiratory tract infections in all age groups [2, 3].

The virus is able to form covalently closed circular (ccc) DNA that is thought to represent episomal structures persisting in the infected cells [4–6]. This assumption could explain the relatively high percentage of asymptomatic HBoV-positive patients in addition to those with primary infection, as the virus persists in a productive but subclinical state [7, 8]. Because it was shown previously that parvoviruses are able to integrate into the host genome [9, 10], and especially parvovirus B19 is associated with several cancers [11–18], colorectal and lung cancer tissues were analyzed with regard to the presence of HBoV-DNA and were found positive in about 20% of the samples [19]. In addition, we previously reported two confirmed cases of usual interstitial pneumonia (UIP) and fibrosis associated with infection of HBoV [20]. Apparently, HBoV appears to persist by forming a cccDNA comparable to the persisting cccDNA of the human hepatitis B virus. Together, all facts suggest a similar mechanism as observed for HBV, starting with acute infection, leading to chronic infection or persistence, leading to fibrosis, maybe resulting in tumours. This infection model resembles chronic HBV-courses frequently resulting in hepatocellular carcinomas [21–23]. Although many processes that lead from fibrosis to the formation of carcinoma in progression of the HBV infection are not yet fully understood, from cell culture studies, animal models, and clinical observation it turned out that mainly the innate immune response to the chronic viral infection play a major role in the cancerogenesis of the HBV-induced tumours [23]**.** Given the fact that animal models are still lacking for the investigation of the HBoV infection, the basis for HBoV research is the analysis of clinical cohorts and the usage of specialized cell culture models, in which at least some parts of the immune response can be investigated in order to elucidate the complex pathophysiology of HBoV infections. Considering that HBoV was found in bronchoalveolar lavages (BALs) of patients with fibrosis and the fact that there are special cytokines regulated during the process of fibrosis [24–26], we analyzed BALs from HBoV patients regarding their cytokine expression. The cytokine patterns observed in those patients were then compared to the originate innate immune response from CuFi-8 cells, a human cell line which is permissive for HBoV when being cultivated as a pseudostratified airway epithelia [27, 28]. Furthermore, the patient cohort includes an unique clinical case in which HBoV-infection was acquired or reactivated between two episodes of BAL sampling.

## Results

The study was divided into two major parts. In the first part of the project, the cytokine patterns in BALs of patients chronically infected or colonized with HBoV were retrospectively analyzed, while in the second part of the project, the patients’ cytokine patterns were compared to the innate immune response during a standardized HBoV infection of cultured human cells and extracted to an HBoV specific cytokine expression pattern.

The BALs were archived for routine purposes and stored at -80°C before they were subjected to the current analyses. Before being archived, BALs were routinely screened cytologically and displayed a broad range from normal cellular distributions to neutrophilia, but no distinct cellular pattern specific for an HBoV infection could have been identified (Table 1).

**Table 1:** Overview of the patients basic characteristics including differential cytology of the BAL fluid, protein content in the BAL, and the infection status of human herpes viruses and human bocavirus. Also given is the number of the Western spot blot belonging to an individual patient presented in S1 Fig.

Patient	Sex	Age	cell counts/ml BAL				Protein (mg/ml BALF)	HBoV	HHV	HBoV copies per ml BAL	Blot-Nr. (S1 Fig)
			lymphocytes	alveolar macrophages	neutrophiles	eosinophiles					
**1**	m	54	2	18	127	0	0,37	/	EBV	0	I
**2**	f	34	2	230	22	0	0,05	/	/	0	II
**3**	m	61	3	5	111	27	0,29	/	/	0	III
**4**	f	62	10	17	302	4	0,29	/	/	0	IV
**5**	m	28	3	158	101	0	0,03	/	CMV	0	V
**6**	m	73	280	182	5	0	10,89	/	/	0	VI
**7**	m	55	1	23	127	2	0,09	/	/	0	VII
**8**	m	32	6	140	10	0	<0,024	/	/	0	VIII
**9**	f	58	5	1	210	0	0,59	/	/	0	IX
**10**	m	69	30	320	20	5	0,13	/	/	0	X
**11**	m	51	48	397	8	0	<0,024	/	EBV	0	XI
**12**	m	79	7	62	31	1	0,18	/	/	0	XII
**13**	m	58	16	77	2	17	0,03	+	-	3.36 x 10^6^	XIII
**14**	m	74	6	91	12	0	12,53	+	EBV	2.31 x 10^6^	XIV
**15**	m	62	21	340	12	0	15,63	+	-	5.05 x 10^6^	XV
**16**	m	75	5	8	103	0	0,04	+	-	1.62 x 10^3^	XVI
**17**	m	61	0	39	270	0	0,19	+	EBV	5.96 x 10^5^	XVII
**18**	f	64	1	25	6	0	<0,024	+	-	3.74 x 10^5^	XVIII
**19**	f	59	33	161	137	7	0,22	+	EBV	4 x 10^5^	XIX
**20**	m	61	5	15	72	0	0,09	+	EBV	6.41 x 10^6^	XX
**21**	f	39	19	361	2	1	0,07	+	/	8.99 x 10^5^	XXI
**22**	f	43	3	106	104	0	0,09	+	/	3.56 x 10^6^	XXII
**23**	m	72	1	245	4	0	<0,024	+	/	1.85 x 10^5^	XXIII
**24**	f	75	19	34	290	0	0,18	+	EBV	4.12 x 10^6^	XXIV
**25**	m	72	9	12	360	16	0,09	+	/	3.06 x 10^5^	XXV
**26**	m	39	0	8	110	0	13,17	+	+	3.07 x 10^5^	XXVI
**27**	f	73	9	208	47	9	0,08	+	HSV-1	6.2 x 10^6^	XXVII
**28**	f	40	3	15	24	33	0,85	+	-	4.18 x 10^5^	XXVIII
**29**	f	40	79	163	11	0	0,22	+	HSV-1	2.2 x 10^5^	XXIX
**30**	m	78	7	88	380	0	0,1	+	-	2.59 x 10^7^	XXX
**31**	m	68	2	43	9	0	0,09	+	EBV	1.567 x 10^6^	XXXI
**32**	f	66	11	140	37	1	0,04	+	EBV	8.34 x 10^6^	XXXII

All BALs were routinely tested for all relevant respiratory pathogens, but except for the (non-causative) detection of persisting herpes viruses (HHV), no pathogen other than HBoV was detected in the HBoV cohort. The HHV detections in the HBoV-cohort were due to persistence but not to acute infections. In the control cohort no respiratory virus was detected, but three patients were detected with persisting human herpesvirus detection (Table 1).

**Fig 1:**
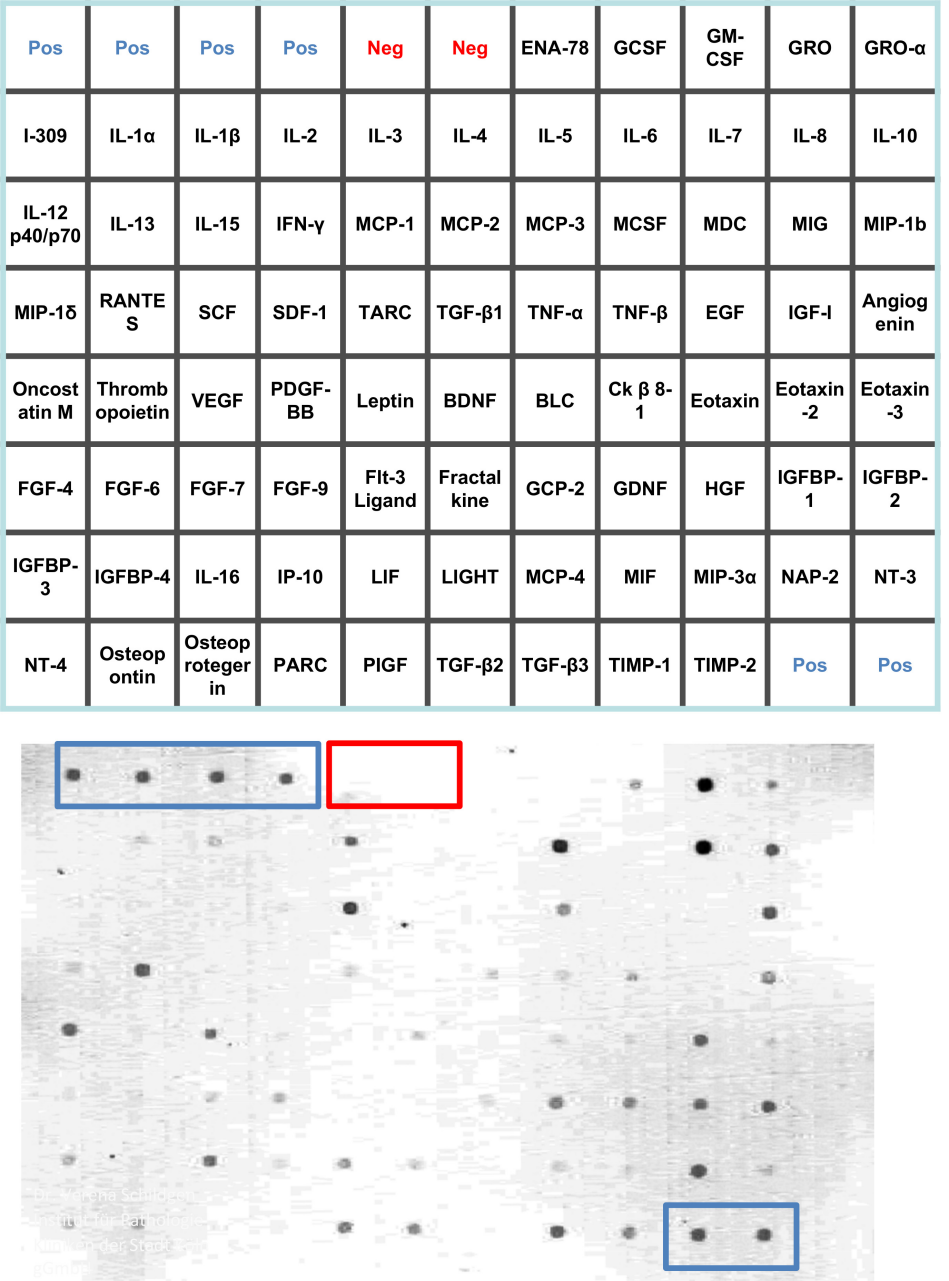
Overview of the cytokine panel and the locations of the 80 cytokines including the internal asssay controls and representative spot blot. Blue letters and boxes indicate the positive controls, red letters and boxes show the negative controls.

The patients’ age ranged between 28 and 78 years (mean: 58·59 y; median: 61 y), 20 patients were male, 12 were female. In a retrospective approach the expression of 80 cytokines in 20 HBoV-positive BALFs and 12 HBoV-negative BALFs were determined by semi-quantitative Western spot blot analyses (Fig 1 and S1 Fig).

The Western spot blots were subjected to digital densitometry and the normalization and quantification were performed in relation to the total protein content of the BAL fluid and in relation to the positive and the negative control spots (i.e. background signals). The negative background controls are labelled by red frames in the individual blots, while the positive controls are labelled by blue frames (S1 Fig).

**Fig 2:**
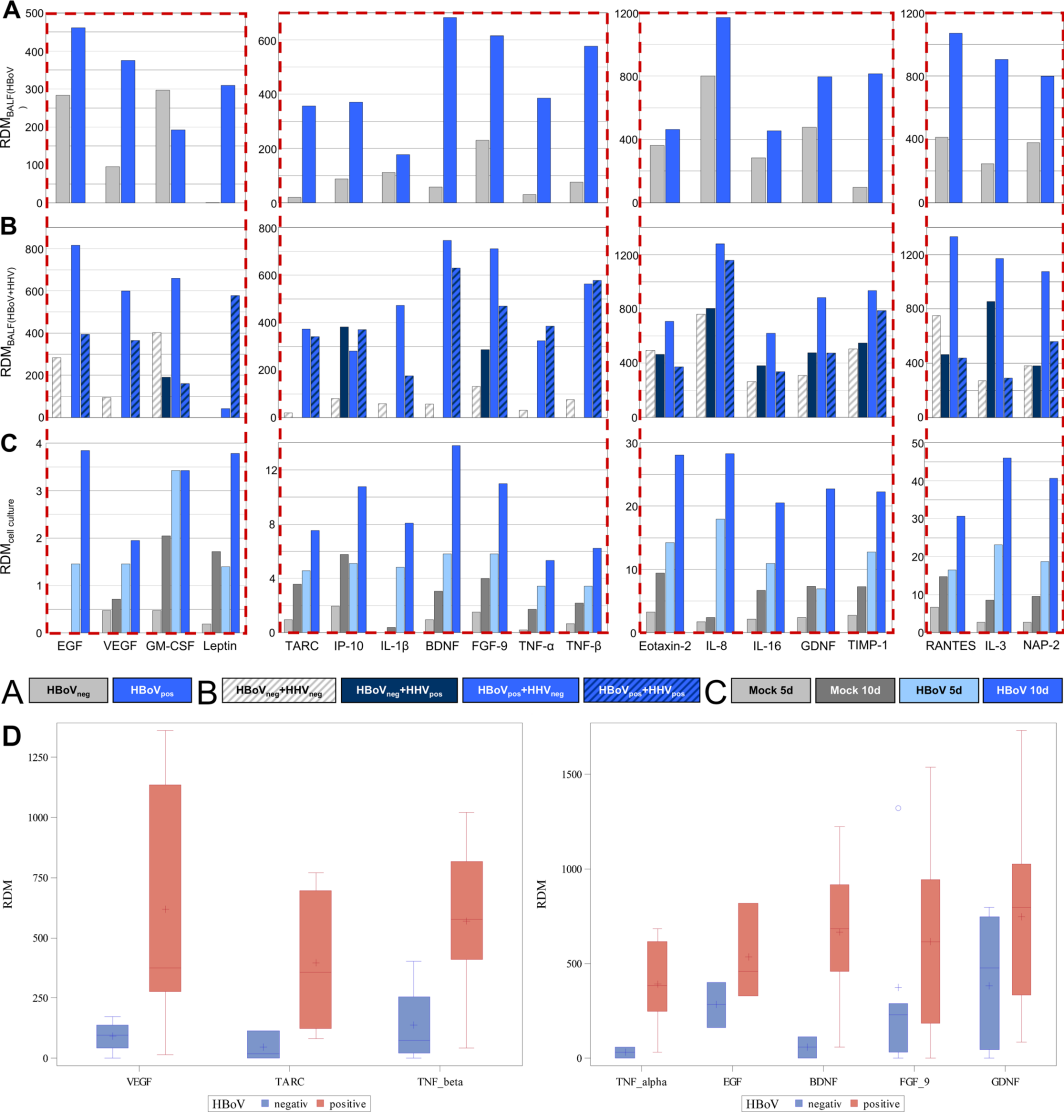
Results of the normalized densitometric quantification of the semiquantitative analyses of the cytokine expression patterns in HBoV positive and negative patients and comparison to HBoV infected and mock infected cell cultures. Panel **A** shows an overview of the entire HBoV positive patient cohort vs. the HBoV negative control group. In panel **B** the HBoV positive group and the control group were divided into HHV positive and HHV negative patients in order to identify HBoV unspecific effects on the expression of cytokines. Panel **C** displays the results of the cell culture experiments. Only those cytokines are displayed that were regulated in the patients/control cohorts. Normalization was performed against the assay internal controls and the total protein content of the respective sample. RDM, relative density measurement. Panel **D** shows SAS boxplots of those parameters, for which a statistical or local significance was identified. The lines in the boxes show the median, crosses and circles indicate the mean.

Cytokine amounts were calculated as relative density measurements (RDM) and are shown in Fig 2.

Although cytokine expression varies between individual patients and within the control group, several cytokines appear to be regulated by the HBoV infection (Fig 2). Cytokines consistently regulated in the HBoV-positive patients include EGF, VEGF, GM-CSF, Leptin, TARC (CCL17), IP10, IL-1β, BDNF, FGF-9, TNF-α, TNF-β, Eotaxin-2 (CCL24), IL8, IL-16, GDNF, TIMP-1, RANTES (CCL5), IL-3, and NAP-2 (CXCL7). All these cytokines are clearly upregulated in the majority of HBoV-positive patients (Table 2) in relation to the negative control group of patients (Fig 2A). In order to exclude an effect from persisting herpes viruses—in some BAL samples DNA of HSV-1, CMV, or EBV was detected—the true HBoV monoinfection cytokine patterns were compared to the cytokine patterns of BALs with a simultaneous detection of herpesvirus DNA (Fig 2B).

**Table 2:** The table presents an overview about all analyzed cytokines, the number of patients, control patients, and respective percentages in which the individual cytokines were present, and the information which cytokines were expressed in patients and cell cultures infected with HBoV.

Cytokine	No. patients		%		Cytokine	No. patients		%		Cytokine	No. patients		%	
	HBoV–	HBoV+	HBoV–	HBoV+		HBoV–	HBoV+	HBoV–	HBoV+		HBoV–	HBoV+	HBoV–	HBoV+
ENA-87	2	3	16,7	15	MIP-1δ (CCL15)	11	14	91,7	70	Fractalkine	2	4	16,7	20
GCSF	1	3	8,3	15	RANTES (CCL5)	12	20	100	100	GCP-2	3	10	25	50
GM-CSF	7	16	58,3	80	SCF	0	7	0	35	GDNF	12	18	100	90
GRO	11	20	91,7	100	SDF-1	0	5	0	25	HGF	7	17	58,3	85
GROα (CXCL2)	1	4	8,3	20	TARC (CCL17)	4	13	33,3	65	IGFBP-1	11	20	91,7	100
I-309	0	0	0	0	TGF- β1	0	5	0	25	IGFBP-2	11	20	91,7	100
IL-1α	10	16	83,3	80	TNF-α	7	14	58,3	70	IGFBP-3	11	20	91,7	100
IL-1β	5	11	41,7	55	TNF-β	5	13	41,7	65	IGFBP-4	2	1	16,7	5
IL-2	1	1	8,3	5	EGF	2	6	16,7	30	IL-16	11	20	91,7	100
IL-3	11	19	91,7	95	IGF-1	1	2	8,3	10	IP-10 (CXCL10)	11	15	91,7	75
IL-4	0	0	0	0	Angiogenin	8	14	66,7	70	LIF	11	19	91,7	95
IL-5	0	1	0	5	Oncostatin	11	19	91,7	95	LIGHT	5	14	41,7	70
IL-6	4	6	33,3	30	Thrombopoet	0	0	0	0	MCP-4	0	0	0	0
IL-7	0	1	0	5	VEGF	6	13	50	65	MIF	5	11	41,7	55
IL-8	12	20	100	100	PDGF-BB	3	7	25	35	MIP-3 α (CCL20)	1	2	8,3	10
IL-10	11	20	91,7	100	Leptin	4	7	33,3	35	NAP-2 (CXCL7)	12	20	100	100
IL-12	1	5	8,3	25	BDNF	7	16	58,3	80	NT-3	2	6	16,7	30
IL-13	1	7	8,3	35	BLC	1	4	8,3	20	NT-4	6	13	50	65
IL-15	1	6	8,3	30	Ck β 8–1	3	11	25	55	Osteopontin	12	20	100	100
IFN-γ	1	5	8,3	25	Eotaxin (CCL11)	5	10	41,7	50	Osteoproteg	0	2	0	10
MCP-1 (CCL2)	10	14	83,3	70	Eotaxin-2 (CCL24)	11	20	91,7	100	PARC (CCL18)	2	8	16,7	40
MCP-2 (CCL8)	0	0	0	0	Eotaxin-3	5	5	41,7	25	PIGF	11	20	91,7	100
MCP-3 (CC7)	0	0	0	0	FGF-4	2	4	16,7	20	TGF-β2	11	18	91,7	90
MCSF	10	19	83,3	95	FGF-6	1	4	8,3	20	TGF-β3	0	4	0	20
MDC (CCL22)	1	2	8,3	10	FGF-7	8	14	66,7	70	TIMP-1	11	19	91,7	95
MIG (CXCL9)	0	0	0	0	FGF-9	12	19	100	95	TIMP-2	10	15	83,3	75
MIP-1b (CCL4)	11	20	91,7	100	Flt3-Lig.	1	3	8,3	15	**n**_**(HBoV-)**_ **= 12; n**_**(HBoV+)**_ **= 20**				

The upregulation of the expression for most cytokines is more pronounced in the HBoV-positive/HHV-negative group than in the HBoV-positive/HHV-positive group. Some cytokines are upregulated exclusively in the presence of HBoV, as in the control group of HHV-positive/HBoV-negative patients those cytokines are not upregulated. This phenomenon is observed mainly for TNF-α, TNF-β, EGF, VEGF, BDNF, TARC (CCL17), and IL-1β, while Leptin is upregulated solely in the group of HBoV/HHV double infected patients, giving rise to the hypothesis that both viruses jointly influence the expression of this cytokine (Fig 2B). The expression of some other cytokines seems to be triggered by an additional HHV-persistence, e.g. IL-8 (CXCL8) and IP-10 (CXCL10), which are similarly upregulated in both patient groups (Fig 2B).

In the group of HBoV positive patients, the VEGF is significantly higher than in the group of HBoV negative patients (p = 0.01). A local significant one sided difference could be shown for TARC (p = 0.037), TNF-beta (p = 0.015), TNF-alpha (p = 0.09), EGF (p = 0.09), BDNF (p = 0.09), FGF-9 (p = 0.06), and GDNF (p = 0.09) (Fig 2D).

No significant difference could be revealed for: TARC/IP-10 ratio, IP-10, GM-CSF, IL-1-beta, IL-3, IL-8, RANTES, Eotaxin-2, IL-16, NAP-2, and TIMP-1. However, it is worth to note that for all cell culture experiments the standard deviation from mean for all cytokines shown in Fig 2 was below 5%, underlining the biological significance.

In the next step we analysed the TARC (CCL17)/IP-10 (CXCL10) ratio in our patient cohort as well as in cell culture, because a study by Kishi and colleagues revealed an elevated TARC (CCL17)/IP-10 (CXCL10) ratio in patients suffering from lung fibrosis. In fact, this ratio is higher in the entire group of HBoV patients vs. the control group (1·41 vs. 0·22) (Fig 2A). Anyway, if single patient ratios were analysed, this effect was less or even absent in some patients, whilst in cell culture this effect was reproducible at day 5 post infection (mock: 0·48 vs. HBoV: 0·89) and at day 10 post infection (mock: 0·62 vs. HBoV: 0·7) (Table 3).

**Table 3:** The TARC (CCL17)/IP-10 (CXCL10) ratio was described as a marker for fibrogenesis in the lung in patient with usual interstitial pneumonia and subsequent idiopathic pulmonary fibrosis. Thus, Table 3 summarizes the TARC (CCL17)/IP-10 (CXCL10) ratios related to the patient cohorts and the cell cultures. The higher the ratio is, the higher is the risk for development of fibrosis.

	TARC (CCL17) _[RDM]_	IP10 (CXCL10) _[RDM]_	TARC (CCL17)/IP10 (CXCL10)ratio	in figure
HBoV_neg_	19.56	88.20	0.22	2a
HBoV_pos_	356.79	252.27	1.41	2a
HBoV_neg_+HHV_neg_	19.56	80.29	0.24	2b
HBoV_neg_+HHV_pos_	0	381.56	0	2b
HBoV_pos_+HHV_neg_	372.67	279.50	1.33	2b
HBoV_pos_+HHV_pos_	340.91	370.37	0.92	2b
CuFi-8 mock 5d	0.95	1.96	0.48	2c
CuFi-8 mock 10d	3.56	5.74	0.62	2c
CuFi-8 HBoV 5d	4.55	5.11	0.89	2c
CuFi-8 HBoV 10d	7.54	10.76	0.7	2c

However, this ratio is not yet an established marker, but it appears, that an altered TARC (CCL17) expression is a relevant event in fibrogenesis [29]. As TARC (CCL17) is not measurably expressed in HBoV negative individuals, but in HBoV positive patients with and without HHV coinfection, this protein appears to be dependent on HBoV infection (Fig 2B). An interesting phenomenon is that GM-CSF appears to be downregulated in the HBoV infection if the entire HBoV-positive patient cohort is analysed (Fig 2A), but if itemized to HBoV-monoinfected and HHV-coinfected patients this downregulation can be ascribed to the influence of HHV coinfections (Fig 2B). However, all observations of cytokine expression levels in patients are snapshots of the individual immunological response to the bocavirus infection and co-pathogens and may be influenced by numerous factors in vivo such as drug based therapy, diet, and disease history, and, most crucial, reflect only a small part of a complex network of interactions between tissues and immune cells and mechanisms. Consequently, it was necessary to analyse the original innate immune response to the human bocavirus infection, i.e. the cytokine expression of the infected cell without presence of additional cell types belonging to the cellular immune response. For this reason, pseudostratified air-liquid interface cultures of CuFi-8 cells were infected with human bocavirus as previously described and the basal cellular medium was collected at distinct time points after infection before being subjected to semiquantitative Western dot blots.

All of the above mentioned cytokines that displayed an increased expression in HBoV-positive patients were also upregulated in HBoV infected cells, both compared the harvesting days 5 and 10 and compared to mock infected cells, respectively (Fig 2C). It is important to mention that EGF, an important growth factor playing a major role in lung cancers, is not measurably expressed in non-infected cells, whilst in HBoV infections the expression leads to strong signals and high RDMs (Fig 2C). It is obvious, that most cytokines accumulate over the observation period of 10 days and thus the overall amount of expressed protein increases, but nevertheless an HBoV specific cytokine expression is significantly increased in infected cells.

Besides the upregulation of cytokine expression in both cell cultures and HBoV infected patients, there are some cytokines that were not detected in any of the patients or in the cell culture media. These cytokines include I-309, IL-4, MCP2, MCP3, MCP4, MIG (CXCL9), and Thrombopoetin, whereas GCSF, IL2, IL5, IL7, IL13, IL15, MDC (CCL22), TGF-β1, IGF-1, BLC, FGF-6, GCP-2, IGFBP-4, MIP-3α (CCL20), PARC (CCL18), TGF-β3 were expressed in some patients but not in cell culture (Table 2).

These data allow elimination of some hypothetical pathways involved in HBoV pathogenesis and it shows that during HBoV infection *in vivo* further components of the innate and cellular immune responses are triggered either individually or independently of HBoV.

**Fig 3:**
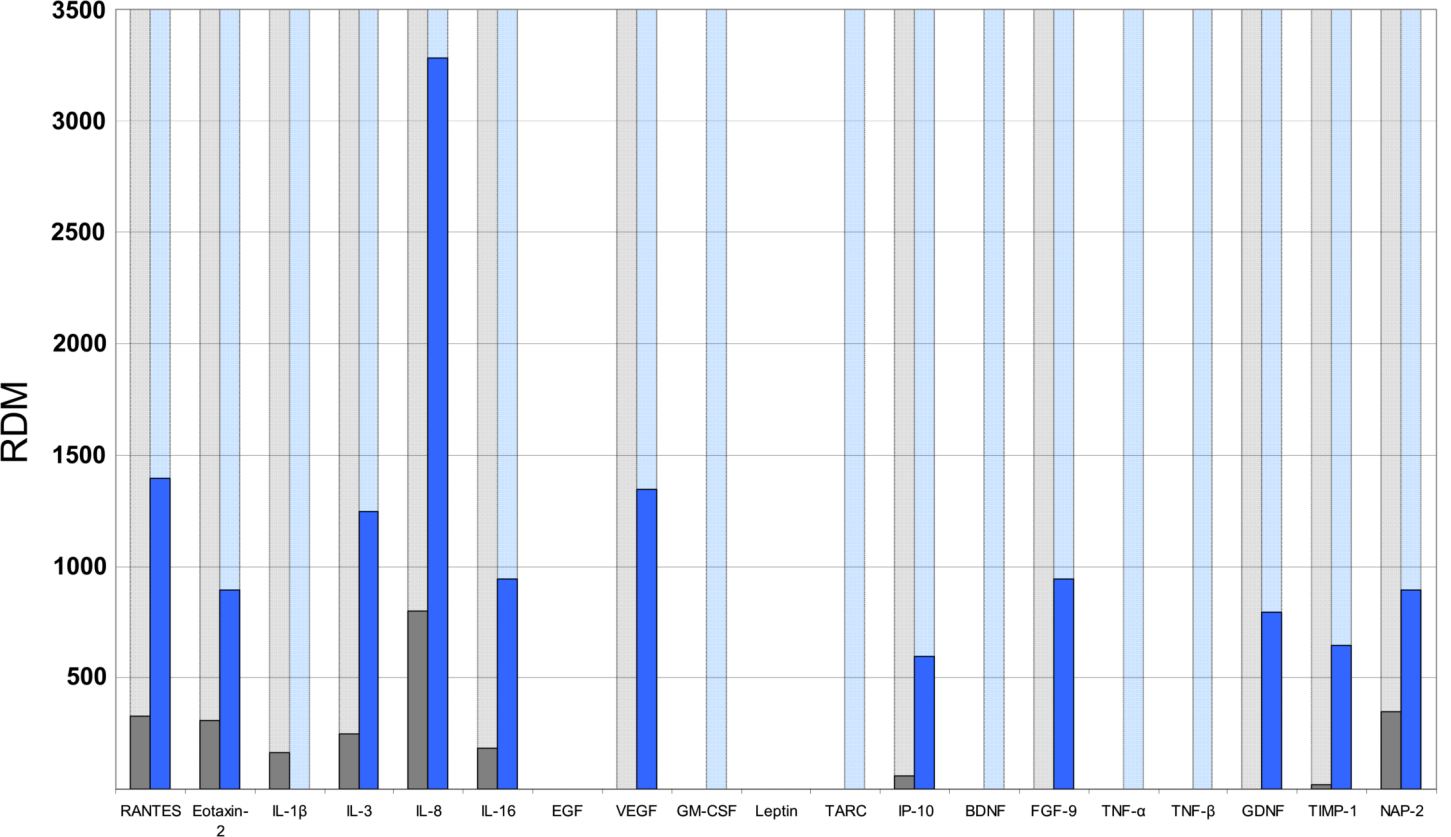
Quantitative analyses of cytokine expression in the clinical case. All analyzed cytokines were quantified as described above, a light grey or light blue column indicates that measurable cytokine expression was observed. The dark grey and dark blue columns indicate the quantified expression of the respective cytokine, grey was the HBoV negative BAL, blue the HBoV positive BAL.

Beyond that, detailed analysis of the included patients revealed a so far unique clinical case: a 61 year old male patient, who was hospitalized because of respiratory symptoms, developed an acute HBoV infection or reactivation during the hospital stay. Unfortunately, no corresponding serum sample was available retrospectively as serology was performed by an external laboratory service provider, which did not archive serum aliquots, whilst BAL was analyzed in our laboratory. Therefore, it cannot be differentiated whether the HBoV detection was a novel infection or due to reactivation of persisting virus. In this particular case archived BALFs before as well as during HBoV infection were available for investigation (Fig 3, and S2 Fig).

This follow up supported the hypothesis that the above described upregulations of cytokine expressions could be correlated with an acute or a conceivably reactivated persisting HBoV infection. MCP-2 (CCL8),-3,-4, MIP-3α (CCL20), Eotaxin-1 (CCL11), MIG (CXCL9), SDF-1, IL-4,-5,-7,-15, EGF, IGF, PDGF-BB, FGF4, FGF6, GCSF, I-309, IL12 p40/p70, IFN-γ, MDC (CCL22), Thrombopoetin, Leptin, BLC, Flt-3 Ligand, IGFBP-4, MIF, NT-4, Osteoprotegerin were not expressed at all, whereas Fig 3 shows that most cytokines expected to be influenced by HBoV are much more pronounced in the HBoV-positive BAL.

Although cytokine levels in the HBoV negative BAL can be assumed higher than in healthy people, because a diagnosed *Pneumococcus* and recurrent infections lasted the whole treatment period, it was set as baseline. Considering that the HBoV positive BAL was taken during a temporary recovery of the patient, it can be supposed that, at least in this patient, cytokines expressed beyond the baseline during HBoV replication are specific for an HBoV infection.

The patient died at last in course of a second flare up of pneumococcal sepsis.

## Discussion

In the majority of clinical studies and single cases described and observed so far the human bocavirus type 1 was associated with mild to severe acute respiratory infections and symptoms ranging from the common cold to life threatening pneumonia, mainly in children but occasionally also in adults [2, 30].

Due to the fact that with the detection of HBoV also multiplexing technologies were introduced in the daily laboratory practice [31–34] and detection of virtually all respiratory pathogens has become an indirect requirement for publication of clinical studies on viral respiratory infection, the human bocavirus is frequently detected together with other pathogens. Because this effect also applies to other respiratory viruses with ability to persist such as Adenoviruses, too [35], it is discussed that HBoV may be a causative pathogen in coinfections rather than a harmless bystander [36]. This discussion will continue as long as no animal model is available due to the narrow host range of the virus, and Koch’s postulates cannot be fulfilled.

However, there is an increasing body of evidence that HBoV not only contributes to harmless respiratory infections such as common cold, but also may persist triggering chronic airway remodelling and fibrosis [19, 20, 27, 37, 38], as well as being involved in cancerogenesis [19, 39].

Because there is no animal model available for the HBoV infection yet, standardized testing of the hypothesis that HBoV could be at least one factor in the complex process of lung fibrogenesis, maybe leading to tumour development, was done in a clinical patient cohort and validated in a cell culture model. We analysed the cytokine profiles of patients with BAL tested positive for HBoV and compared these observations to standardized cell culture experiments.

Lung fibrosis, especially the idiopathic lung fibrosis (IPF) is characterized by a Th2-type dominated immune response in the affected tissue (reviewed by: [24–26]). The Th2 response in the lung is accompanied by increased expression levels of IL-4, IL-5, IL-10, and IL-13 and is followed by increased levels–besides others–of CCL17 (TARC (CCL17)) and CCL5 (RANTES). Moreover, fibrosis is accompanied by increased expression of TNF and IL-8 (CXCL8), and it is worth to mention that the neutralization of TARC (CCL17) leads to a reduction of fibrosis in an animal model [25, 40]. In addition, an elevation of the TARC (CCL17)/IP-10 (CXCL10) ratio is also characteristic for fibrosis and was previously discussed as a marker for IPF [29].

In the present study, exactly those cytokines and chemokines that are associated with Th2 response, lung fibrosis, and subsequent lung cancer were upregulated dependent of HBoV presence but independent of additional herpesviruses present in the BAL fluid. Moreover, a so far unique follow up case included in our study, in which the infection or reactivation of HBoV occurred between two episodes of BAL sampling, and in which the fibrosis associated cytokines were expressed in association with the HBoV infection but not before, supports the data obtained for the total patient cohort. This leads to the conclusion, that HBoV colonisation/chronic infection may be at least one trigger that could stimulate airway remodelling. However, it could be argued that *in vivo* not only the resident airway epithelial cells are involved in the immune response but also additional patient specific factors could contribute to altered profibrotic cytokine profiles. In order to address this problem we performed cell culture experiments in an air-liquid-interface culture of human airway epithelial cells. These experiments confirmed that the profibrotic cytokines were expressed by the infected cell cultures but were hardly or not at all expressed in mock-infected cells and they reveal that the identified cytokines belong to the initial immune response after HBoV infection.

Thereby, also in cell culture, the TARC (CCL17)/IP-10 (CXCL10) ratio showed a tendency of profibrotic patterns, although in the individual patients this effect was less obvious than in the entire cohort. In cell culture Leptin expression was dramatically increased in infected vs. mock-infected cells, what is a remarkable observation as Leptin was shown to increase the expression of collagen and lead to fibrosis in the liver [41]. This observation in turn would fit to the hypothesis that the cccDNA of HBoV in analogy to the cccDNA of HBV may cause a subclinical chronic inflammation sometimes leading to fibrosis. In the patient cohort Leptin was almost entirely expressed in HBoV/HHV positive patients maybe indicating that HHV is able to suppress mechanisms, which *in vivo* prevent an elevated Leptin expression. Another example for an indirect stimulation of fibrotic processes is the expression of GM-CSF. Patients, who did not or only at a low level express GM-CSF, maybe belong to a risk group for fibrosis development, because GM-CSF is necessary for the PGE2 (prostaglandine E2) synthesis in mice [42], which in turn inhibits the release of IL-1β and TNF-α from macrophages [43].

A limitation of our study is the fact that in the statistical analyses the situation of multiple hypotheses was neglected and only unadjusted p-values were presented for descriptive reasons only. To consider all the test hypothesis correctly, an adjustment of the p-values (for example a Bonferoni correction) would be necessary. But, with the limited number of patients, the relatively high variation of values and the enormous amount of cytokines it is nearly impossible to detect a statistically significant difference considering the multiple hypothesis situation, which was the major reason to confirm the biological significance of our observations in the fully controlled cell culture model. Taking the adjustment of p-values into account, a much greater patient cohort would be necessary to detect some cytokines that are significant different between the HBoV groups, but economically the cost-benefit balance of such a study is questionable.

Furthermore the two populations (HBoV positive and HBoV negative) may be quite different not also in their cytokines. Because of the limited number of patients and the high costs for testing 80 cytokines in a single patient, a group-balancing such as matching does not make any sense. It would be quite interesting to repeat this study in a much greater cohort and to balance for at least some baseline characteristics.

The hypothesis that the HBoV infection is indeed a trigger for fibrogenesis is confirmed by some more parameters that showed an increased expression in the HBoV positive patient cohort and also in cell culture: TIMP-1 that is also increased in liver fibrosis [44]; and NAP-2 (CXCL7) that was found to be higher expressed in kidney fibrosis [45], while neutralization of NAP-2 (CXCL7) reduces lung fibrosis in the animal model [46]. The HBoV infection is associated with an enhanced expression of different growth factors as VEGF, FGF-9 and EGF. VEGF activates the ERK1/2 pathway, which is involved in angiogenesis and inflammation processes in the lung [47] and it is thought to enhance fibrogenesis in the lung, too [48]. Furthermore it came out that attenuation of the VEGF and FGF-9 pathways reduces fibrosis [49], consequently a higher expression promotes fibrogenesis. The same applies to EGF, which plays an important role in the development of liver fibrosis and its inhibition also leads to a reduction of fibrosis [50]. According to VEGF and FGF-9 elevated EGF expression supports fibrogenesis and the subsequent development of cancer. Not surprisingly, lung fibrosis goes ahead with an increased risk of lung cancer of 30% and higher compared to non-fibrotic patients (reviewed by [51]).

Besides VEGF TNF-α is overexpressed in early stages of pulmonary fibrosis [52] and high TNF-α titers are furthermore correlated with a higher risk of developing a hepatic periportal fibrosis [53] and stimulate the collagen synthesis in fibroblasts [54]. Also in the case of TNF-α neutralization of the protein reduces fibrogenesis as shown in animal experiments with mice and rats with previously induced fibrosis [55, 56]. Additionally, patients as well as the cell culture show an increase in IL-1β expression, which in murine lung epithelial cells enhances TNF surface expression [57]. A further remarkable cytokine heavily expressed in HBoV positive samples is BDNF. As the above mentioned proteins BDNF activates pathways leading to fibrosis and is associated with IPF [58, 59], but in addition increased BDNF levels were detected in patients suffering from COPD (chronic obstructive pulmonary disease) [60] and were correlated with a poorer prognosis in NSCLC (non small cell lung cancer) patients [61]. RANTES (CCL5), which is also substantially increased in HBoV-positive patients and cell cultures, contributes to fibrosis by inducing the expression of collagen-1 [62]. Interestingly the dysregulation of IL-16, which also could contribute to fibrosis, is more pronounced in HBoV infected CuFi-8 cells compared to the data from the patient cohort, an effect that could be attributed to the cell line itself. CuFi-8 cells are derived from a patient with cystic fibrosis, and IL-16 has been shown to be dysregulated in this disease [63]. A further cytokine that requires foremost attention, although only marginally elevated in HBoV positive patients but threefold higher in infected cell cultures compared to mock controls, is Eotaxin-2 (CCL24). It is not only more expressed in fibrosis [64] but also in colorectal cancers [65] and our group detected HBoV-DNA in about 20% of analysed colorectal tumour samples (Schildgen et al., 2013). In short the overall cytokine expression patterns observed in our study strongly resemble the profiles observed in different phases of lung fibrosis and typically appear especially for UIP and IPF [66] and furthermore, many of the HBoV specific expressed cytokines are also involved in lung cancer and tumour progression [61, 67–76].

It is important to mention, that besides the co-detected herpesviruses, also chronic underlying diseases such as COPD or sarcoidosis could have influenced the cytokine patterns and thus could have led to a bias in our study. However, given the fact that in the cohort of control patients only one sarcoidosis case was confirmed, while none occurred in the HBoV-infected cohort, this is rather unlikely. Di Stefano [77] and coworkers have e.g. shown, that CCL5/RANTES is upregulated in COPD patients, and also in our cohort such an upregulation was obvious. However, this high “background” could be also associated with the fact that the majority of the patients suffered from signs of airway infections that in such cases RANTES (CCL5) is upregulated intrinsically.

Regarding the human herpes viruses it can be excluded that the HBoV-specific effects we mention have been influenced by human herpesviruses. Although it was described, that human herpesviruses such as CMV induced the expression of cytokines such as CCL-5 and IP-10 (CXCL10) [78, 79], we have observed that the effects were present also in the HHV-negative patients. It is, however, important to keep in mind that HHV infection appear to interact with HBoV, as there were co-effects we cannot clearly show how this interaction appears. For this reason, coinfection studies in cell cultures would be a challenge for future studies.

A further observation that requires discussion is the fact that the single patient we describe in detail on the one hand shows an upregulation of most cytokines that are influenced by the HBoV infection in vitro, but even not all cytokines regulated in vitro were observed also in the course of the case. This can, however, be easily explained. This patient had acquired a pneumococcal sepsis and therefore displayed another background cytokine profile. In vivo, much more factors influence the cytokine expression, including the cellular immunity that is not present in the CuFi-8 cell cultures. It is worth to note that despite this background, HBoV triggered the profibrotic cytokine pattern in the patient, thus it is worth to get more in detail in future studies. The case moreover is unique so far, as there has been not even a single report in which the results of two follow up BAL sampling episodes were reported, whilst the patient acquired HBoV between these samplings.

Taken together, the present study gives rise to the hypothesis that the human bocavirus is indeed a trigger for pulmonary fibrosis. The data derived from the total patient cohort are confirmed by a single case in which for the first time the onset of an acute or reactivated HBoV infection could be observed by conversion of the HBoV status in the BAL fluid. Further confirmation comes from the control trials in CuFi-8 cells that additionally show that the fibrogenesis and the Th2 type response are not only initiated by the immunocompetent cells present in the lung but from the resident airway epithelia cells.

Nevertheless, our study has the bias that we could show that HBoV may be a trigger of fibrosis, but we were not able to show how many fibrosis patients have an active HBoV infection, are HBoV colonized, or have undergone an HBoV infection previously. Due to the retrospective character of our study, no corresponding sera were available as the primary sampling of BALs was not intended to screen for active HBoV infection, but instead HBoV was identified as the only respiratory pathogen in the patients, in a subcohort accompanied by human herpesviruses (EBV and CMV). Taking into account the high seroprevalence of >95% [80–83] it is most likely that virtually all patients with lung fibrosis will be seropositive, but a systematic study addressing this topic is missing so far. Consequently the next step should be the screening of fibrosis patients to get an overview of the HBoV prevalence in this patient group. Nevertheless, we have already shown that HBoV could be detected in approximately 20% of lung and colorectal cancers [19]. Considering that this may reflect the tip of the iceberg, there are many patients in whom at least the persisting HBoV as a fibrosis promoting and maybe even carcinogenic factor could be avoided by an antiviral therapy or vaccination in the future.

Moreover, the majority of patients with HBoV infections will not be hospitalized nor will receive a BAL sampling, and the minority of HBoV positive patients receiving BAL sampling will have been subject to a preceding BAL sampling immediately before the onset of the HBoV infection. Thus, the data on the pathogenesis of HBoV infection will remain limited and rely on clinical observations, as long as no animal model is available for the study of the HBoV infection. At present, clinical data and case reports are the best source of information of the *in vivo* course of the HBoV infection in its natural environment, which is the HBoV-infected lung. Therefore, in concert with the recent literature, the present study is instructively and a good indicator for the direction of future studies, not at least as the hepato-cellular carcinoma induced by the hepatitis B virus infection is the result of a similar cascade of episomal viral persistence, immune reactions, fibrosis, and progression to cancer. Finally, it must be concluded, that although the HBoV infection may not be the single cause of lung fibrosis and cancers, it appears to be an important player in the pathogenesis of these clinical entities that requires more attention in the development of chronic airway diseases.

## Material and Methods

### Consent and Ethical Statement

All procedures were performed in accordance with the declaration of Helsinki and according to a vote of the Ethical Committee of the Private University of Witten-Herdecke (vote no. 73/2012). This vote was specifically approved for the current study. Due to the retrospective nature and the double-blinded patient samples used in the study, the Ethical Committee concluded that no written informed consent was required. Consequently, the Ethical Committee waived the need for written informed consent. The study cohort consisted exclusively of adult patients.

### Patient samples

20 HBoV positive samples and 12 HBoV negative samples tested by PCR were randomly selected from archived bronchoalveolar lavages from our routine clinical laboratory (Table 1). In the control group, one patient had a sarcoidosis as an underlying disease, whilst none of the other control patients had any chronic lung disease such as COPD, asthma, or any other diagnosed chronic disorder. One patient suffered from a tracheal stenosis, one from chronic cough, four patients had a confirmed pneumonia, the rest suffered from a suspected airway infection. In the HBoV positive patient cohort, only one patient suffered from chronic asthma and experienced an exacerbation likely due to an airway infection, one had an allergic alveolitis, one a pneumonia. Moreover, 3 patients suffered from chronic cough, 2 from acute cough, 2 from ARDS, and two from partial respiratory insufficiency, while the rest suffered from respiratory symptoms likely to be caused by respiratory infections.

All samples were analyzed with the RespiFinder Smart22 and the MeningoFinder Smart7 Customized Assay (Pathofinder, NL) being able to detect 18 viral and 4 bacterial respiratory pathogens and 9 neurotropic viruses with facultative lung tropism. In detail, the detectable pathogens were: influenza viruses A and B incl. H1N1, RSV A and B, HMPV, HBoV, PIV1-4, HCoV-NL63, -OC43,-229E, and HKU-1, adenoviruses, Legionella pneumonia, Chlamydia pneumonia, Bordetella pertussis, and Mycoplasma pneumoniae. Patients were negative for P. jirovecii by PCR, for Aspergillus by galactomannan assays (BioRad, Munich, Germany), and for Mycobactrium tuberculosis by PCR/hybridization (Chipron, Berlin, Germany) or by anamnesis and bronchoscopy. Exotic viruses and respiratory pathogens like SARS, MERS, Hantavirus pulmonary syndrome etc. were excluded by anamnesis. Further bacterial infections were excluded by conventional routine microbiological culturing. Samples were stored at -80°C. The study was performed retrospectively. All clinical samples were collected in 2012 and 2013.

### Cell lines and primary cells

Human embryonic kidney 293 (HEK293) cells were obtained from American Type Culture Collection (ATCC via LGC Standards, Wesel, Germany), and were cultured according to the instructions provided by the supplier. CuFi-8 cells originate from the Tissue and Cell Culture Core, Center for Gene Therapy, University of Iowa and were a kind gift of Aloysius Klingelhutz, John Engelhardt, and Phil Karp (University of Iowa, USA). CuFi-8 were immortalized from cystic fibrosis human primary airway cells, and were cultured as described previously [27].

### Human airway epithelium (HAE) cultures

CuFi-8 cells differentiate on collagen-coated, semipermeable membrane inserts (0·6 cm^2^, Millicell-PCF; Millipore, Billerica, MA) into an air-liquid interface culture. After 3–4 weeks of culture in DMEM:F12 medium (50%:50%) containing 2% Ultroser G (Pall BioSepra, Cergy-Staint-Christophe, France) the polarity of the HAE was determined based on the transepithelial electrical resistance (TEER) using an epithelial Volt-Ohm Meter (Millipore). HBoV1 infection was performed at minimal values of 600–800 Ω per insert [27].

### Virus infection

Virus was produced and quantified essentially as described earlier by Huang and coworkers [27]. In brief, cell culture experiments were performed in duplicate in an „air-liquid-interface”culture of differentiated CuFi-8 cells as previously described by Huang and coworkers [27]. These were infected with 10^5^ Bocavirus particles released from HEK293 cells after transfection of the full length HBoV1 plasmid pIHBoV1 [27] from the apical surface for 5h followed by aspiration of the virus and three washes with PBS to remove unbound virus. Cells were harvested 5 or 10 days post infection, respectively. As mock controls differentiated CuFi-8 HAEs were incubated for 5h with supernatant of PUC18 transfected HEK293 cells. Untreated differentiated CuFi-8 HAEs were used as negative controls.

### Cytokine expression

Cytokine profiles induced by HBoV-infection in CuFi-8 cell culture supernatant and in bronchoalveolar lavage fluid (BALF) of 20 HBoV-positive and 12 HBoV-negative patients were analysed by semi-quantitative Western spot blot analyses. For cytokine expression analysis the Cytokine Human Membrane Antibody Array ab133998 (Abcam, Cambridge, UK) was used, which was performed predominantly according to the manufactures instructions. Samples were always incubated over night at 4°C and were washed with the larger amount of buffer. Detection was carried out through biotin conjugated antibodies, HRP conjugated Streptavidin and 1 ml DAB (Abcam, Cambridge, UK). Evaluation was made by densitometry with normalization to positive (PC) and negative (NC) controls, as well as to overall protein concentration of the respective sample. This process of normalization led to the value of RDM (relative densitometric measurement). Protein concentration was determined using the Pierce Assay (Thermo Scientific, Germany). The method detects the following cytokines: ENA-78, GCSF, GM-CSF, GRO, GRO-alpha (CXCL2), I-309, IL-1alpha, IL-1beta, IL-2, IL-3, IL-4, IL-5, IL-6, IL-7, IL-8 (CXCL8), IL-10, IL-12 p40/p70, IL-13, IL-15, IFN-gamma, MCP-1 (CCL2), MCP-2 (CCL8), MCP-3 (CC7), MCSF, MDC (CCL22), MIG (CXCL9), MIP-1beta (CCL4), MIP-1delta (CCL15), RANTES (CCL5), SCF, SDF-1, TARC (CCL17), TGF-beta1, TNF-alpha, TNF-beta, EGF, IGF-I, Angiogenin, Oncostatin M, Thrombopoietin, VEGF-A, PDGF-BB, Leptin, BDNF, BLC, Ckß8-1, Eotaxin (CCL11), Eotaxin-2 (CCL24), Eotaxin-3 (CCL26), FGF-4, FGF-6, FGF-7, FGF-9, Flt-3 Ligand, Fractalkine, GCP-2, GDNF, HGF, IGFBP-1, IGFBP-2, IGFBP-3, IGFBP-4, IL-16, IP-10 (CXCL10), LIF, LIGHT, MCP-4, MIF, MIP-3 alpha (CCL20), NAP-2 (CXCL7), NT-3, NT-4, Osteopontin, Osteoprotegerin, PARC (CCL18), PLGF, TGF-beta2, TGF-beta3, TIMP-1, TIMP-2

### Statistical Analysis

Statistical analyses were performed in order to identify cytokines regulated in the patient cohort. The biological significance of the regulation of cytokines was tested in cell culture. As starting hypothesis we focused on VEGF, a marker known to be associated with lung fibrosis. Therefore this is the only parameter were the statistical significant difference between HBoV positive and negative patients was tested (one sided median test for difference in location). A p-value less than 0.1 was considered to be statistically significant.

Not taking into account the multiple-test situation, the non-adjusted p-values and related 90%-confidence intervals for all other parameters were presented for descriptive reasons only. Also for these parameters a one sided median test for difference in location was used. Here, p-values less than 0.1 were considered to be locally significant. The analysis was conducted with the statistical software SAS, version 9.4.

## Supporting Information

S1 Fig Scanned Western Spot blots from patients with and without infection with human bocavirus. Blue frames indicate the assay-internal positive controls; red frames indicate the assay-internal negative controls. Panel **a** shows the HBoV positive patients, panel **b** represents the HBoV negative patients, and panel **c** represents the Western spot blots from CuFi-8 cell cultures fluids of HBoV and mock infected polarized cells at days 5 and 10.
(PDF)

S2 Fig The blots III and XX from S1 Fig belong to a unique clinical case in which the patient was HBoV-negative in the first BAL but became HBoV positive thereafter and viral DNA was detected in the second BAL a few days later. Most likely, this phenomenon is based on a reactivation as no personnel to patient transmission could be identified and as the internal Hygiene barriers were high. Both BALFs were negative for MCP-2 (CCL8),-3,-4, MIP-3^α^ (CCL20), Eotaxin-1 (CCL11), MIG (CXCL9), SDF-1, IL-4,-5,-7,-15, EGF, IGF, PDGF-BB, FGF4, FGF6, GCSF, I-309, IL12 p40/p70, IFN-γ, MDC (CCL22), Thrombopoetin, Leptin, BLC, Flt-3 Ligand, IGFBP-4, MIF, NT-4, and Osteoprotegerin. The regulated cytokines are explicitly mentioned, a blue frame indicates the assay internal positive controls, a red frame indicates the assay internal negative controls.
(PDF)
