# Supplementary material for: Lung Infection by Human Bocavirus Induces the Release of Profibrotic Mediator Cytokines In Vivo and In Vitro
Source: PLoS One. 2016 Jan 25;11(1):e0147010. doi: 10.1371/journal.pone.0147010 (PMC4726461; doi:10.1371/journal.pone.0147010)

supplemental figure 1a

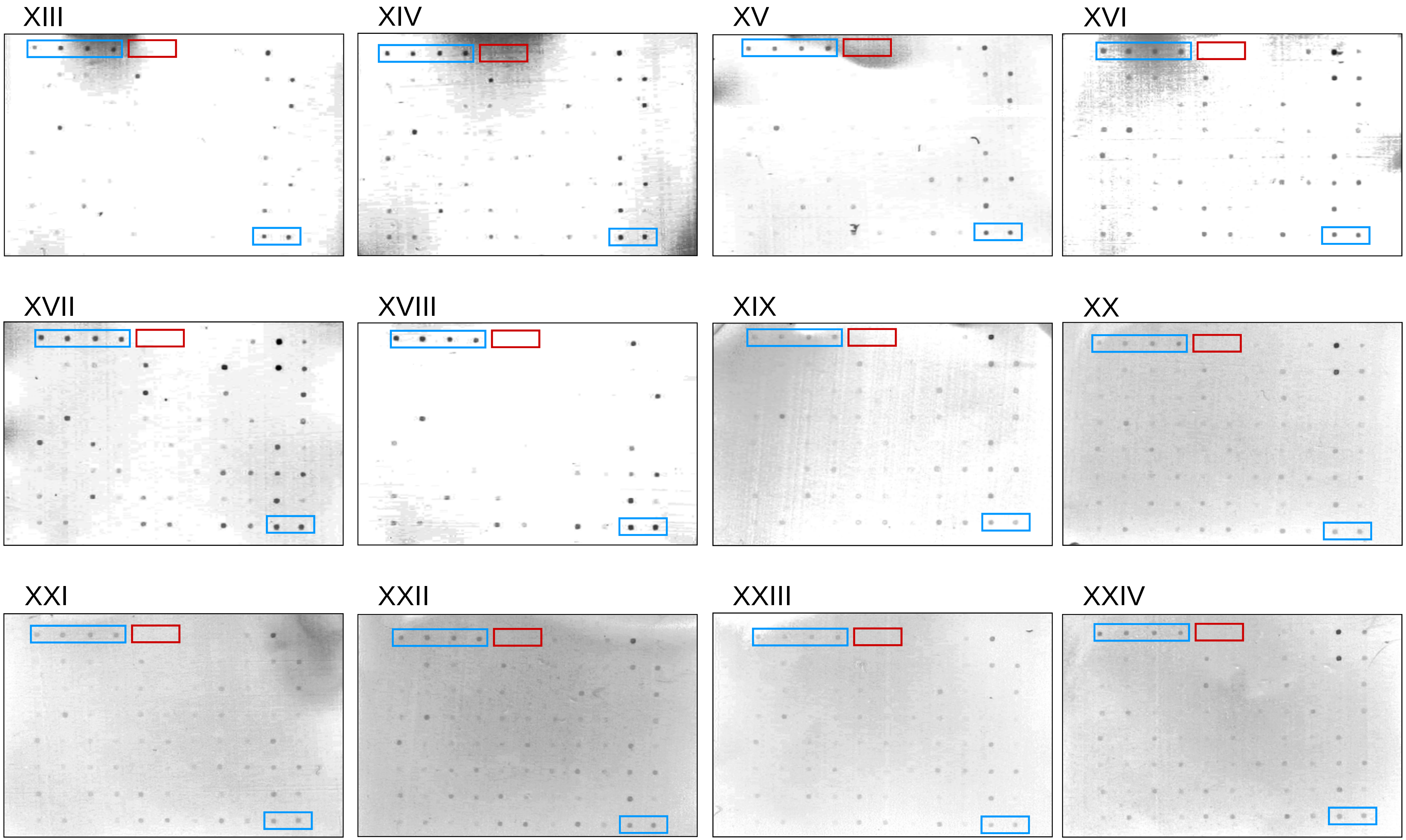

Supplemental figure 1a, continued

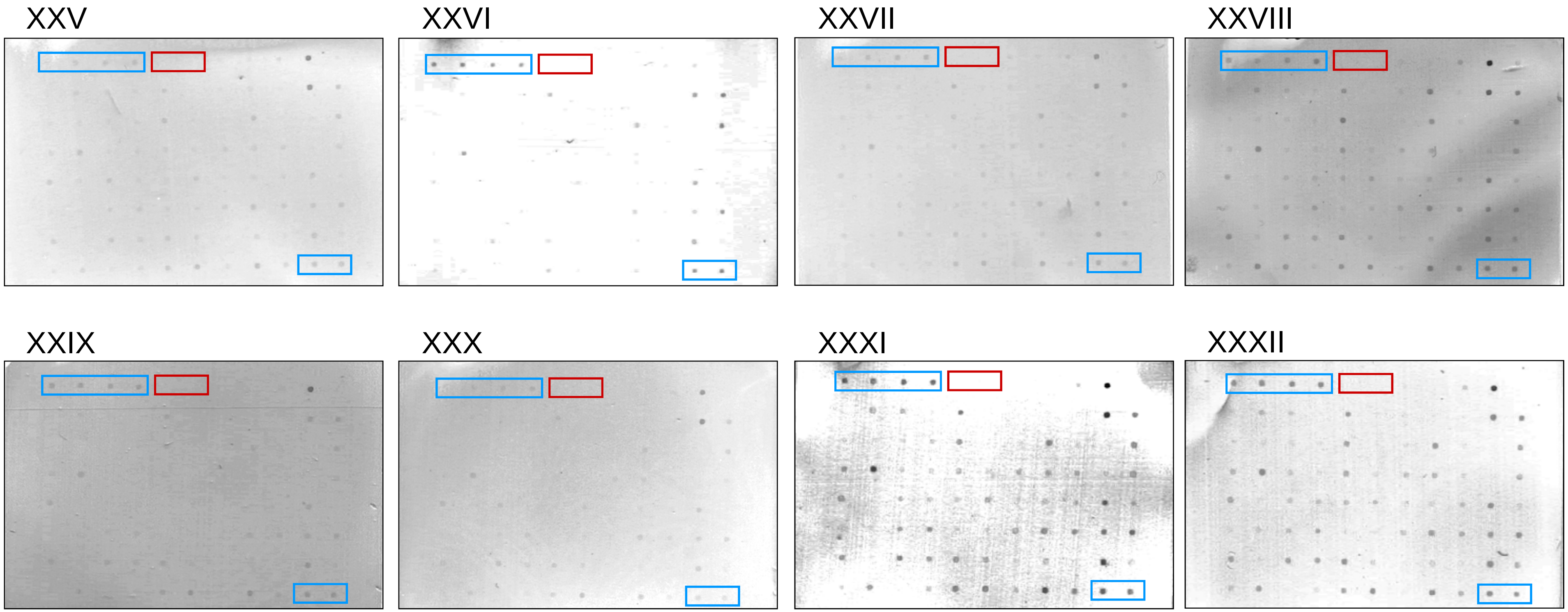

supplemental figure 1b

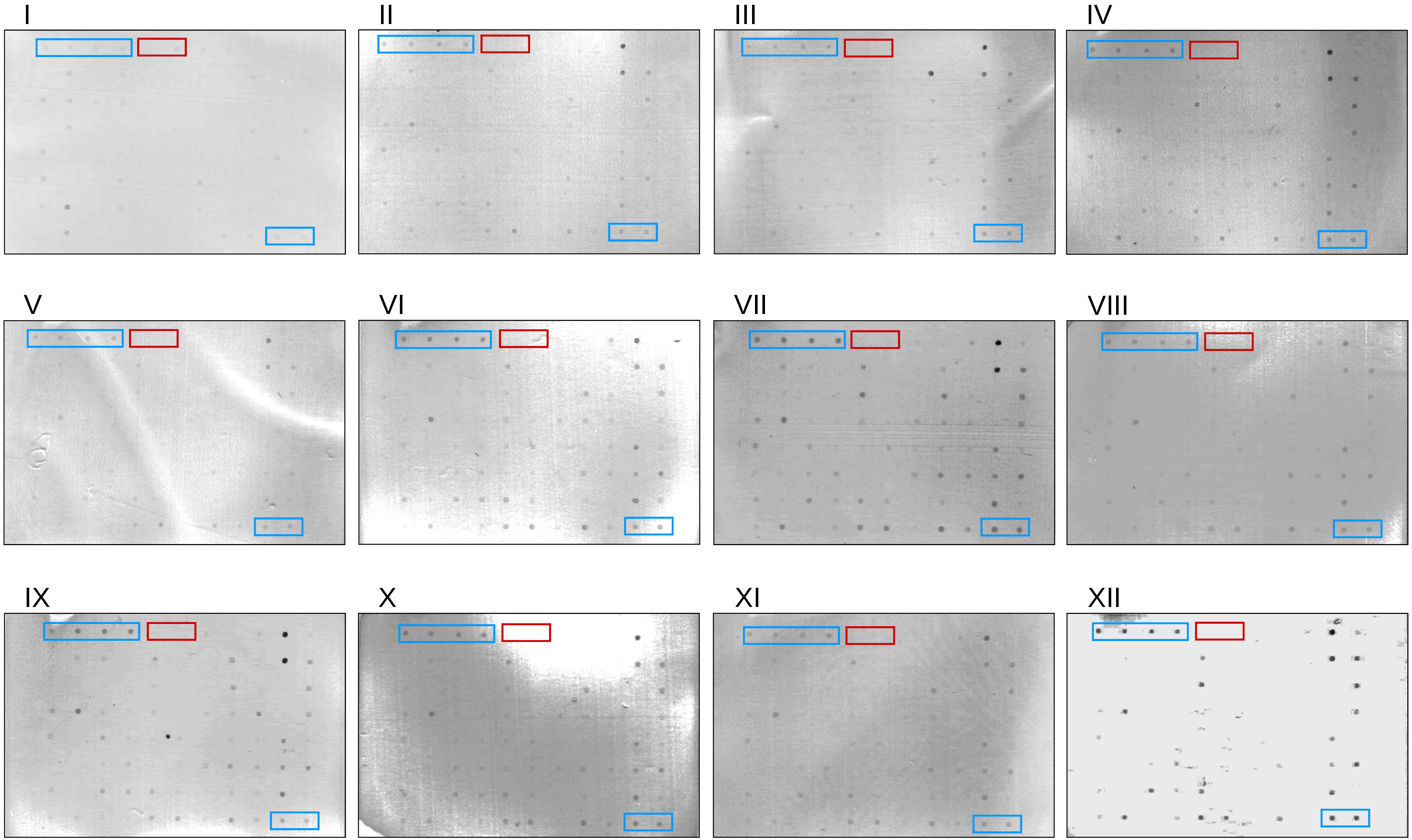

supplemental figure 1c

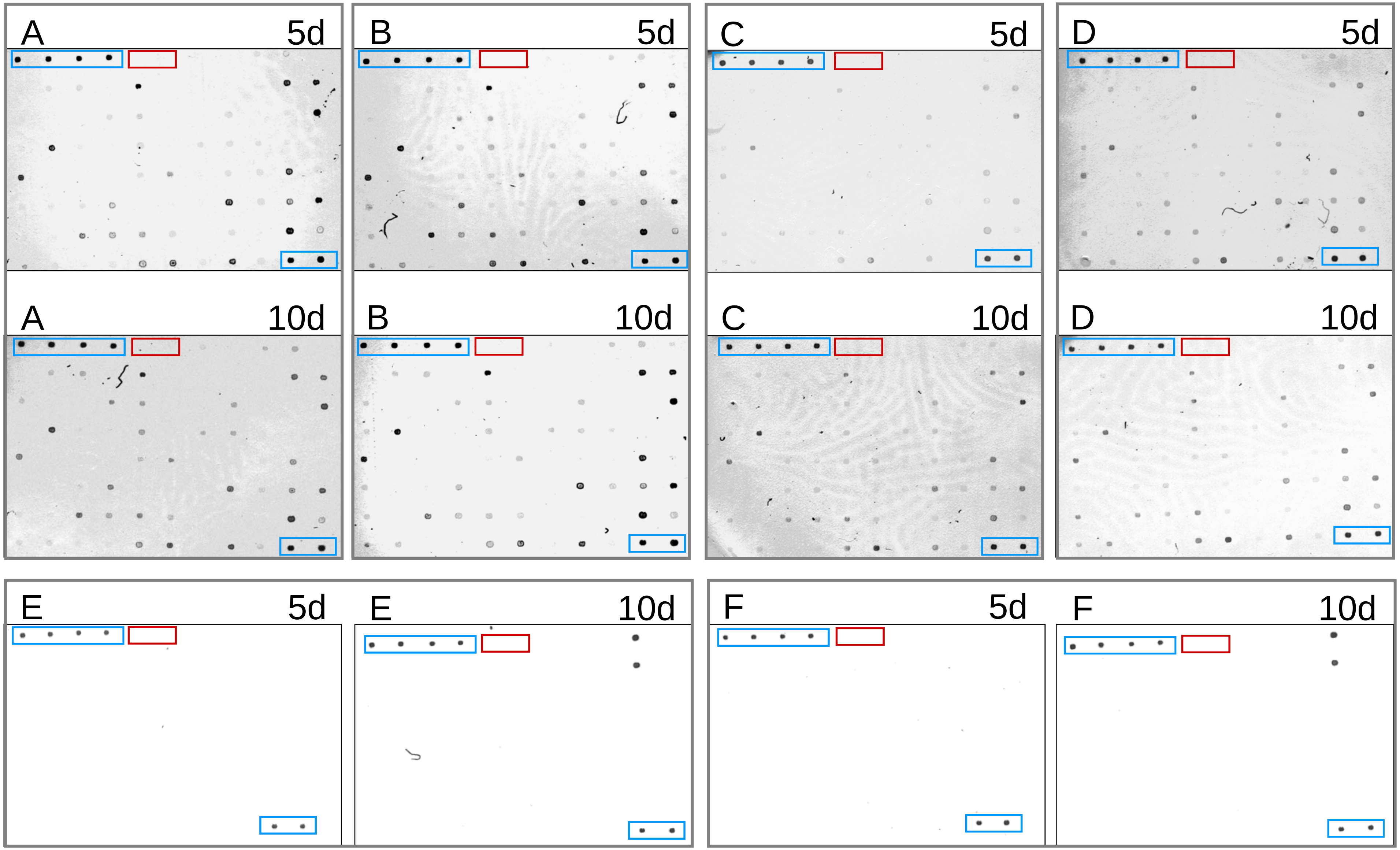

Supplement: S1 Fig — Blue frames indicate the assay-internal positive controls; red frames indicate the assay-internal negative controls. Panel a shows the HBoV positive patients, panel b represents the HBoV negative patients, and panel c represents the Western spot blots from CuFi-8 cell cultures fluids of HBoV and mock infected polarized cells at days 5 and 10. (PDF) [file pone.0147010.s001.pdf]
