# Supplementary material for: Lung Infection by Human Bocavirus Induces the Release of Profibrotic Mediator Cytokines In Vivo and In Vitro
Source: PLoS One. 2016 Jan 25;11(1):e0147010. doi: 10.1371/journal.pone.0147010 (PMC4726461; doi:10.1371/journal.pone.0147010)

Supplemental figure 2

Blot III from supplemental figure 1 →HBoV negative

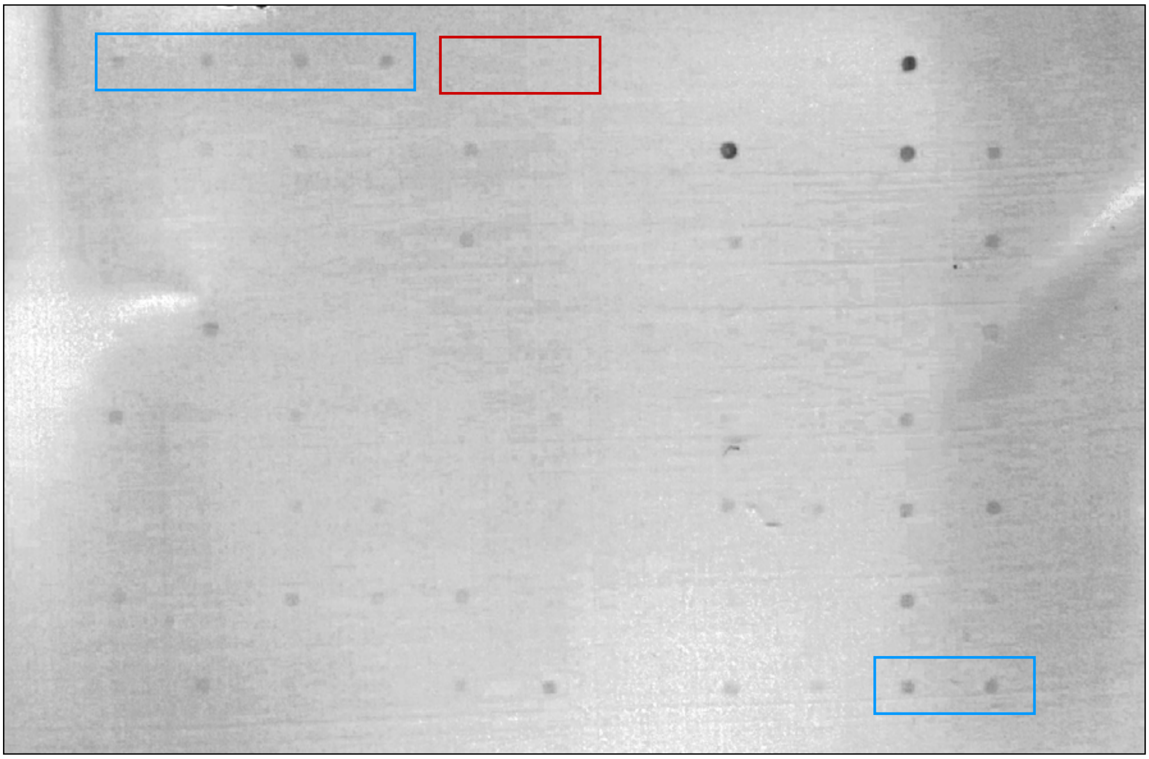

Blot XX from supplemental figure 1a ⟶ HBoV positive

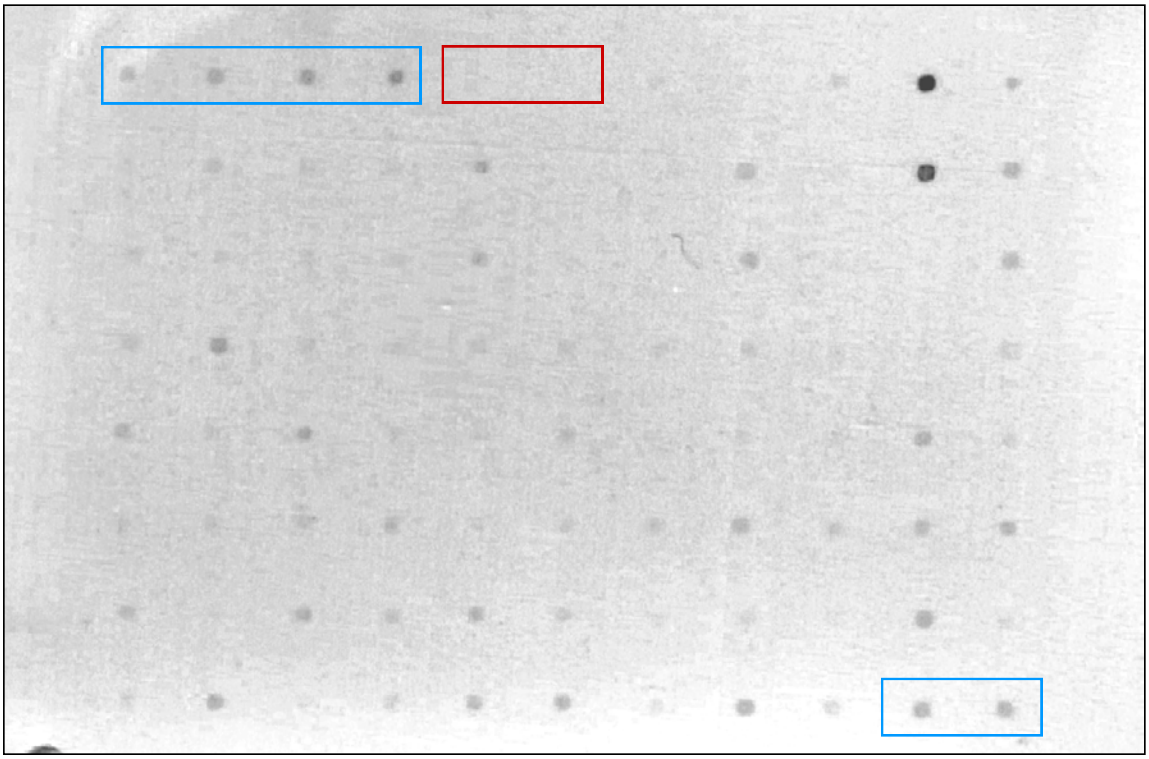

Supplement: S2 Fig — Most likely, this phenomenon is based on a reactivation as no personnel to patient transmission could be identified and as the internal Hygiene barriers were high. Both BALFs were negative for MCP-2 (CCL8),-3,-4, MIP-3α (CCL20), Eotaxin-1 (CCL11), MIG (CXCL9), SDF-1, IL-4,-5,-7,-15, EGF, IGF, PDGF-BB, FGF4, FGF6, GCSF, I-309, IL12 p40/p70, IFN-γ, MDC (CCL22), Thrombopoetin, Leptin, BLC, Flt-3 Ligand, IGFBP-4, MIF, NT-4, and Osteoprotegerin. The regulated cytokines are explicitly mentioned, a blue frame indicates the assay internal positive controls, a red frame indicates the assay internal negative controls. (PDF) [file pone.0147010.s002.pdf]
